# Induction of Expression of CD271 and CD34 in Mesenchymal Stromal Cells Cultured as Spheroids

**DOI:** 10.1155/2018/7357213

**Published:** 2018-07-31

**Authors:** Bruno Corrêa Bellagamba, Patrícia Bencke Grudzinski, Pedro Bins Ely, Paulo de Jesus Hartmann Nader, Nance Beyer Nardi, Lindolfo da Silva Meirelles

**Affiliations:** ^1^Laboratory for Stem Cells and Tissue Engineering, PPGBioSaúde, Universidade Luterana do Brasil, Canoas, RS, Brazil; ^2^Plastic Surgery Service, Irmandade Santa Casa de Misericórdia de Porto Alegre, Porto Alegre, RS, Brazil; ^3^Pediatrics and Neonatology Service, Hospital Universitário ULBRA Mãe de Deus, Canoas, RS, Brazil

## Abstract

Cultured mesenchymal stromal cells (MSCs) are cells that can be used for tissue engineering or cell therapies owing to their multipotency and ability to secrete immunomodulatory and trophic molecules. Several studies suggest that MSCs can become pericytes when cocultured with endothelial cells (ECs) but failed to use pericyte markers not already expressed by MSCs. We hypothesized ECs could instruct MSCs to express the molecules CD271 or CD34, which are expressed by pericytes in situ but not by MSCs. CD271 is a marker of especial interest because it is associated with multipotency, a characteristic that wanes in MSCs as they are culture expanded. Consequently, surface expression of CD271 and CD34 was detected in roughly half of the MSCs cocultured with ECs as spheroids in the presence of insulin-like growth factor 1 (IGF-1). Conversely, expression of CD271 and CD34 was detected in a similar proportion of MSCs cultured under these conditions without ECs, and expression of these markers was low or absent when no IGF-1 was added. These findings indicate that specific culture conditions including IGF-1 can endow cultured MSCs with expression of CD271 and CD34, which may enhance the multipotency of these cells when they are used for therapeutic purposes.

## 1. Introduction

Cultured mesenchymal stromal cells (MSCs) are cells that exhibit properties such as adherence to plastic, differentiation into mesodermal cell lineages, proliferation, and secretion of trophic factors [1, 2]. Firstly isolated from the bone marrow [3], MSCs have been obtained from several organs and tissues such as the tendon [4], pancreas [5], liver [6], synovial membrane [7], umbilical cord [8], and adipose tissue (AT) [9]. This ubiquitous distribution of cells able to give rise to cultured MSCs is explained by their association with the perivascular niche [10, 11]. Accordingly, MSCs have been proposed to arise from perivascular cells called pericytes [10, 12, 13].

Pericytes are a subset of mural cells that have important roles in blood vessel morphogenesis and remodeling [14], as they interact with endothelial cells (ECs) through the basal membrane and stabilize blood vessels [15]. The relationship between pericytes and MSCs has been initially discussed by Bianco and Cossu [16]; later, this relationship was extended with the suggestion that MSC-like cells originate from pericytes that were activated after tissue injury [10]. More recently, cultured pericytes and MSCs from AT were found to have an essentially identical surface molecule profile (including various pericyte-related markers) and an almost identical gene expression profile, which confirms that pericytes can give rise to cultured MSCs [17].

In addition to their capacity of differentiation into several cell types and their ability to secrete bioactive molecules [1], MSCs have been shown to exhibit angiogenic properties [18], which further suggests their relationship with the perivascular niche. Cocultivation of MSCs with ECs is an effective way to produce vascular structures in vitro to provide prevascularized constructs for tissue engineering [19]. Consequently, a large number of reports have demonstrated the ability of MSCs to interact with ECs to form vascular structures both in vitro and in vivo [20–28].

While the studies on MSC-EC interactions mentioned above focused on the application of MSCs in tissue engineering, only a few of them focused on the molecular phenotype of MSCs cocultured with ECs, and some of these suggest MSCs may assume a native pericyte (nPC) phenotype (i.e., a molecular phenotype characteristic of PCs in their native perivascular niche in vivo) based on the expression of some pericyte-related molecules. Loibl et al. [29] found that direct cell contact between MSCs and ECs upregulates the expression of genes that encode the pericyte-related molecules CD146, NG2 (neural/glial antigen 2), *α*-SMA (*α*-smooth muscle actin), and PDGFR (platelet-derived growth factor receptor) in MSCs. Other studies suggest that MSCs acquire a smooth muscle cell phenotype when in contact with ECs [30, 31].

Detection of the acquisition of an nPC phenotype by MSCs when they are cocultured with ECs is difficult because of some factors. Firstly, cultured MSCs are known to express pericyte-related markers (e.g., PDGFR*β*, NG2, and *α*-SMA) [15] prior to contact with ECs. Secondly, since a number of studies have described pericyte markers after expansion of blood vessel-derived cells in culture, many of these markers may be not specific to nPCs [32]. Consequently, there is no consensus on the most reliable marker for the detection of an nPC phenotype [33, 34].

In view of the above, the use of molecules known to be expressed by pericytes in situ but lost after their expansion in vitro could represent a more strict approach to detect the acquisition of an nPC phenotype by cultured MSCs. CD34, for example, has been detected on the surface human AT nPCs [17, 35] but not on their culture-expanded progeny [17]. Likewise, CD271 has been detected in noncultured pericytes from the human bone marrow [36, 37], kidneys [38], and AT [17], and its expression is lost after culture expansion [17]. In mesenchymal cells, expression of CD271 is intrinsically associated with a primitive phenotype. Esteve et al. found that CD271 expression in CD45^−^CD34^+^CD31^−^ cells obtained from human AT decreased as the freshly isolated cells underwent adipogenic differentiation in vitro [39]. The expression of genes specific to AT stem/progenitor cells was detected in human AT-derived pericytes knowingly positive for CD271 [40]. Human dental pulp-derived MSCs derived from CD271^+^ cells are more clonogenic than their CD271^−^ counterparts, and forced expression of the gene that codes for CD271 in the murine multipotent cell line C3H10T1/2 inhibited their differentiation into osteoblasts, adipocytes, chondrocytes, and myocytes [41].

In this work, we sought to assess the expression of CD34 and CD271 in human AT-derived MSCs cocultured with ECs to check if this physical interaction could stimulate MSCs to take up an nPC phenotype. Consequently, we found that an impressive proportion of MSCs could express CD271 or CD34 in vitro owing to the culture conditions used rather than the physical contact with ECs.

## 2. Materials and Methods

### 2.1. Reagents and Materials

Reagents used in this study were purchased from Sigma-Aldrich Brasil Ltda (São Paulo, SP, Brazil), unless specified otherwise. Fetal bovine serum (FBS) was purchased from Gibco® (Life Technologies do Brasil Com. Ind. Prod. Biotec. Ltda, Itapevi, SP, Brazil). Plasticware used was supplied by Greiner Bio-One Brasil Produtos Medicos Hospitalares Ltda (Americana, SP, Brazil).

### 2.2. Isolation and Culture of Adipose Tissue-Derived Mesenchymal Stromal Cells

Human adipose tissue was obtained as discarded material from liposuction surgeries at Irmandade Santa Casa de Misericórdia de Porto Alegre (ISCMPA) after informed consent of patients. AT from four different donors were used for the isolation of MSCs. This study was approved by the Institutional Review Board of ISCMPA (CAAE 11903713.7.0000.5335).

AT-derived MSCs (ATMSCs) were isolated as previously described [17]. Briefly, AT fragments were mixed with Hank's balanced salt solution (HBSS) supplemented with 10 mM of HEPES in 50 mL centrifuge tubes and centrifuged at 600 ×g for 10 minutes. Floating AT was removed, transferred to a 50 mL centrifuge tube containing an equal volume of collagenase solution (300 units of collagenase I (Worthington Biochemical Corp., Lakewood, NJ, USA)) per mL of Dulbecco's Modified Eagle's Medium-low bicarbonate containing 2% fetal bovine serum (DMEM-LB/2), and incubated at 37°C for 40 minutes in a water bath with intermittent swirling of the tubes. A detailed description of all media formulations used in this study is shown in Table 1. At the end of the incubation period, the contents of the tubes were centrifuged at 250 ×g for 10 minutes. The pellets were mixed and resuspended in 10 mL of red blood cell lysis solution (155 mM NH_4_Cl, 10 mM KHCO_3_, 0.1 mM EDTA, and 1 mM sodium pyruvate in ultrapure water). After incubation for 10 minutes, the tube was filled with DMEM containing 10% FBS (DMEM/10) and centrifuged. The supernatant was discarded, and the pellet was resuspended in DMEM/10. After washing by centrifugation, the pellet was resuspended in DMEM/10, and viable cells were counted using trypan blue in a Neubauer's chamber.

After enzymatic disaggregation, 15,000 cells/cm^2^ were seeded into 75 cm^2^ culture flasks in DMEM/10. Cells were maintained in an incubator at 37°C with a humidified atmosphere containing 5% CO2. When cultures were 80% confluent, cells were collected after incubation with cell detachment basal solution (HBSS containing 1 mM sodium pyruvate and 0.5 mM EDTA) followed by cell detachment solution (cell detachment basal solution containing 0.025% trypsin) and seeded at a density of 4000 cells/cm^2^ into new 75 cm^2^ culture flasks in DMEM/10. At the end of the second passage, ATMSCs were frozen in FBS containing 10% (*v*/*v*) dimethyl sulfoxide or expanded as required for the experiments. On some occasions, ATMSCs were additionally cultured in pericyte medium (PCM, Table 1) for at least 7 days prior to analysis or experiments. The types and concentrations of growth factors in PCM, inclusive IGF-1, were the same as in the proprietary pericyte medium sold by ScienCell Research Laboratories (Carlsbad, CA). Cells between passages 4 and 6 were used in the experiments.

### 2.3. Isolation and Culture of Human Umbilical Vein Endothelial Cells (HUVECs)

Human umbilical cords were obtained as discarded material after full-term deliveries at Hospital Universitário ULBRA Mãe de Deus (Canoas, RS, Brazil) after informed consent from the parturients. The obtainment of umbilical cords has been approved by the Institutional Review Board of ULBRA (CAAE 55076816.2.0000.5349).

Collection of HUVECs was carried out as previously described [42], with slight modifications. Umbilical cords from three different donors were used for the isolation of HUVECs. The umbilical cord vein was washed twice with phosphate-buffered saline (PBS), and a collagenase solution (150 units of collagenase I per mL of DMEM-LB/2) was inserted through one of the vein's ends. The vein's ends were clamped, and the vein was incubated for 10 minutes at 37°C in a water bath. After incubation, the collagenase solution containing HUVECs was removed from the vein and centrifuged at 300 ×g for 10 minutes. Pellets were resuspended in endothelial cell medium (ECM, Table 1) and seeded into 75 cm^2^ culture flasks previously coated with 1% gelatin solution. ECM composition was modified from a previously published study [43], as addition of IGF-1, heparin, ascorbic acid, and hydrocortisone improved HUVEC growth. Cells were maintained in an incubator at 37°C with a humidified atmosphere containing 5% CO2. When cultures were 80% confluent, cells were collected following the same procedure used for ATMSCs. At the end of the second passage, HUVECs were subjected to immunophenotyping and expanded according to the experiments' requirements or frozen in FBS containing 10% of dimethyl sulfoxide. Cells between passages 4 and 6 were used in the experiments.

### 2.4. Culture of Cells as Spheroids

ATMSCs and HUVECs were cultured as spheroids as previously described [44], with some modifications. Briefly, ATMSCs and HUVECs were collected from culture flasks, counted, mixed at a ratio of 2 : 3 (ATMSCs : HUVECs) or added alone (only ATMSCs) to 2 mL propylene tubes, and centrifuged at 150 ×g for 5 minutes. A total of 500,000 cells were used to form the spheroids. We carried out five culture strategies: (i) ATMSCs cultured in DMEM/10 and then as spheroids for 7 days. Cells from two different donors were used in two independent experiments; (ii) ATMSCs precultured in MCDB/2 for 7 days and then cultured as spheroids in MCDB/2 for 7 additional days. Cells from two different donors were used in two independent experiments; (iii) ATMSCs precultured in PCM for 7 days and then cultured as spheroids in PCM for 7 additional days. Cells from three different donors were used in four independent experiments; (iv) ATMSCs precultured in IGF-1-free PCM (IF-PCM) for 7 days and then cultured as spheroids in IF-PCM for 7 additional days. Cells from two different donors were used in two independent experiments; (v) ATMSCs precultured in PCM for 7 days and then cocultured with HUVECs as spheroids in ECM for 7 additional days. Cells from two different donors were used in two independent experiments.

### 2.5. Flow Cytometry

ATMSCs cultured in DMEM/10 in culture flasks (i.e., two-dimensional (2D) conditions), in PCM in 2D conditions or as spheroids (alone or cocultured with HUVECs) as mentioned above, were immunophenotyped by flow cytometry. Cells cultured in 2D conditions were harvested using trypsin-EDTA, and cells from the spheroids were dissociated using type I collagenase. After cell collection and centrifugation, 1 × 10^5^ cells were resuspended in 100 *μ*L of PBS containing 1% bovine serum albumin and were stained with 0.1 *μ*g of fluorochrome-labeled antibody for 30 minutes at room temperature in the dark. Antibodies used included fluorochrome-conjugated anti-human CD11b, CD31, CD34, CD79a, CD90, CD105, CD106, CD146, CD271, CD140b, and NG2 immunoglobulins. Isotype-matched fluorochrome-conjugated control antibodies or unstained cells were used as negative controls. The antibodies used to detect surface markers in this study, as well as their suppliers, fluorochromes, and clone names, are listed in Supplementary Table 1. After washing with PBS, cells were resuspended into 300 *μ*L of PBS, and fluorescence was read in a BD Accuri™ C6 flow cytometer (Becton, Dickinson and Company, Franklin Lakes, New Jersey, USA). The cell populations were identified based on forward-scatter and side-scatter parameters, and 10,000 events were gated for analysis. Flow cytometry histograms and graphics were generated using BD Accuri C6 Software.

### 2.6. Differentiation Assays

Adipogenic differentiation and osteogenic differentiation were performed on ATMSCs cultured in DMEM/10 or in PCM. Cells were seeded at a density of 4 × 10^4^ cells per well in 12-well culture plates. For adipogenic differentiation, cells were cultured in DMEM/10 containing 1 × 10^−8^ M dexamethasone and 5 *μ*M rosiglitazone for 7 to 10 days, when this medium was augmented with 5 *μ*g/mL bovine insulin and cells were cultured for additional time (14–17 days). For osteogenic induction, cells were cultured in DMEM/10 containing 1 × 10^−8^ M dexamethasone and 50 *μ*g/mL ascorbic acid 2-phosphate for 10 days, when this medium formulation was augmented with 2 mM *β*-glycerophosphate and cells were cultured for additional two weeks. Lipid-containing vacuoles of adipocytes were stained with Oil Red O and the calcium-rich extracellular matrix secreted by osteoblasts was detected by staining with Alizarin Red S. Images were acquired using an Axio Vert.A1 microscope (Zeiss, Jena, Germany).

## 3. Results

### 3.1. Characterization of ATMSCs Cultured in 2D Conditions

**Figure 1:**
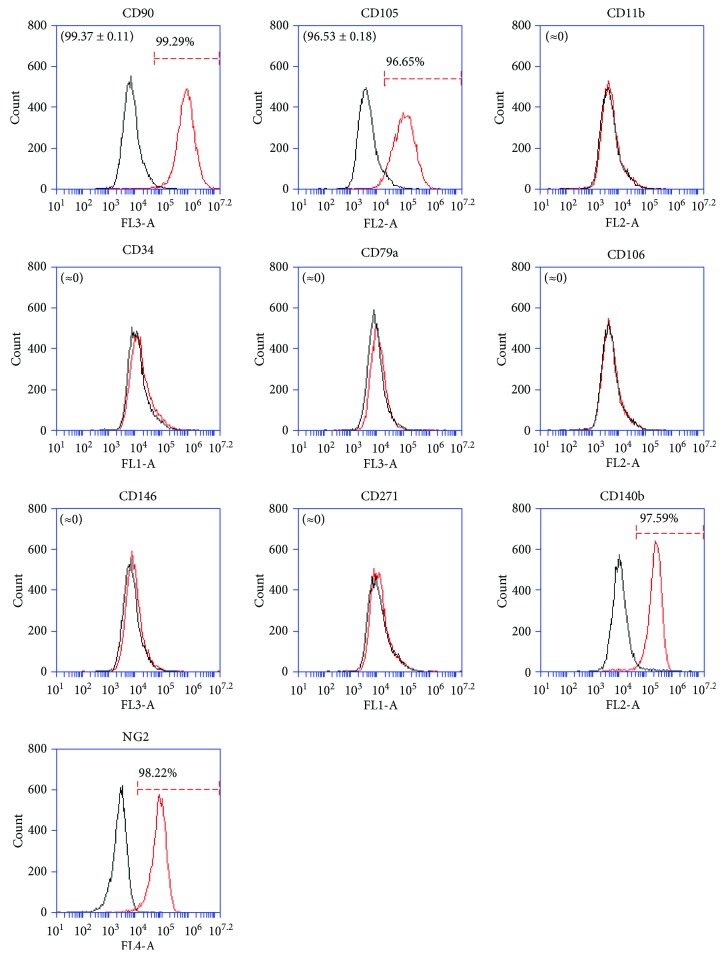
Immunophenotyping of ATMSCs cultured in standard conditions (DMEM/10, in 2D). Cells expressed CD90, CD105, CD140b, and NG2 but lacked expression of CD11b, CD34, CD79a, CD106, CD146, and CD271. Histograms drawn in black represent negative controls. Histograms drawn in red represent the expression of the abovementioned molecules by the cell populations. Overlapping curves represent populations of cells that do not express the tested markers. Histograms are representative of two independent experiments, except for CD140b and NG2, whose data corresponds to a single ATMSC population analyzed. Mean ± standard deviations of the percentage of cells expressing the surface markers are indicated in parenthesis.

**Figure 2:**
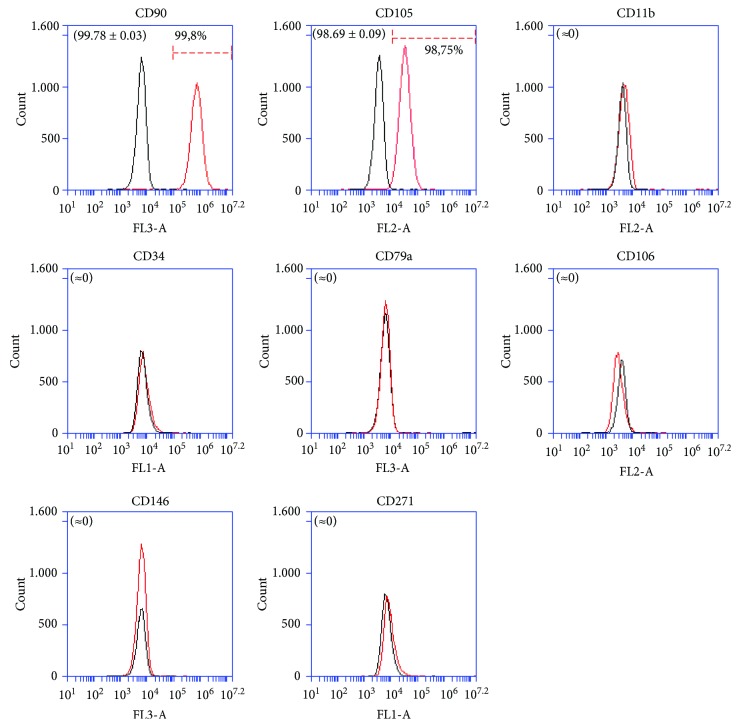
Immunophenotyping of ATMSCs cultured in PCM in 2D. Cells expressed CD90 and CD105 but lacked expression of CD11b, CD34, CD79a, CD106, CD146, and CD271. Histograms drawn in black represent negative controls. Histograms drawn in red represent the expression of the abovementioned molecules by the cell populations. Overlapping curves represent populations of cells that do not express the tested markers. These data are representative of two independent experiments. Mean ± standard deviations of the percentage of cells expressing the surface markers are indicated in parenthesis.

**Figure 3:**
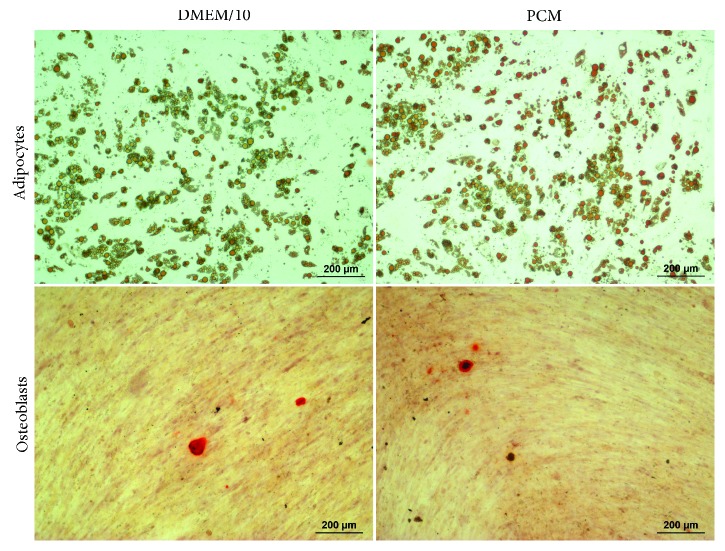
ATMSCs cultured in DMEM/10 or in PCM in 2D were induced to differentiate into adipocytes or osteoblasts. Lipid-containing vacuoles of adipocytes were stained with Oil Red O and the calcium-rich extracellular matrix secreted by osteoblasts was detected by staining with Alizarin Red S.

Firstly, ATMSCs cultured in standard conditions (DMEM/10) and ATMSCs cultured in PCM, both cultured in 2D conditions (culture flasks) for 7 to 10 days, were immunophenotyped. Since ATMSCs cultured in PCM acquire surface molecule and gene expression profiles very similar to those presented by cultured pericytes [17], we decided to verify the expression of the nPC markers CD271 and CD34 on the surface of ATMSCs cultured under pericyte conditions. The flow cytometry analysis showed that ATMSCs have a similar immunophenotype whether cultured in DMEM/10 or PCM. ATMSCs cultured in DMEM/10 (Figure 1) or PCM (Figure 2) do not express CD271 or CD34. Multipotential capacity of ATMSCs cultured in DMEM/10 or PCM was assessed, and cells cultured in both conditions differentiated into adipocytes and osteoblasts, as depicted in Figure 3.

### 3.2. Coculture of ATMSCs and ECs on Matrigel

Since ATMSCs cultured in PCM did not express the nPC markers under study, we assessed the expression of genes that code for CD271, CD34, and NG2 (*NGFR*, *CD34*, and *CSPG4*, resp.) after coculture of ATMSCs with murine ECs (endothelial cell line EOMA) on Matrigel® (Supplementary Materials). In these preliminary experiments, the murine ECs were allowed to develop capillary-like structures onto which ATMSCs were seeded, as physical contact between with these structures was expected to influence the expression of nPC markers on ATMSCs. A murine EC line was chosen to improve the experimental accuracy; the expression of human transcripts was not detected in the murine cells (Supplementary Table 2), which means that only ATMSC-derived *NGFR*, *CD34*, and *CSPG4* would be detected by the probes used. We found increased expression of *NGFR*, *CD34*, and *CSPG4* in ATMSCs cocultured with murine ECs on Matrigel (Supplementary Table 3). Expression of *CD34* by EOMA cells cultured alone in one of the conditions used (culture on Matrigel for 7 days) precluded validation of expression of this gene by ATMSCs alone in this experiment. Conversely, the strategy of using ECs from a different species in cocultures with human ATMSCs was valid for *NGFR* and *CSPG4*, as detection of these transcripts in EOMA cells was absent under all the conditions tested, while it was detectable in ATMSCs. We also found the colocalization of ATMSCs with tubular structures formed by ECs on Matrigel (Supplementary Figure 1), which is a feature of pericytes. Even though this coculture system proved useful for an initial evaluation, we found that only a small number of ATMSCs actually became physically associated with the murine ECs, which could hurdle the detection of the nPC markers under study. Additionally, the quality of capillary-like structures formed by EOMA cells was poor, which called for a different approach. Since the prevailing hypothesis at the time of this experiment was performed was that physical contact with ECs is required for the expression of nPC markers by ATMSCs, the need for increased events of ATMSC-EC contact became evident. Therefore, we sought to establish a three-dimensional (3D) coculture system that could maximize physical contact between ATMSCs and human ECs (HUVECs) with subsequent verification of expression of the nPC markers CD271 and CD34 on the cells' surfaces. The use of intracellular dyes such as CM-DiI or DiO to label the two cell populations, which was used in the preliminary experiments involving coculture of ATMSCs with EOMA cells, was dismissed owing to the possible transfer of these dyes between these two cell types, as intracellular dyes have been shown to be transferred from ATMSCs to other cell types when cultured together [45].

### 3.3. Coculture of ATMSCs and HUVECs as Spheroids

**Figure 4:**
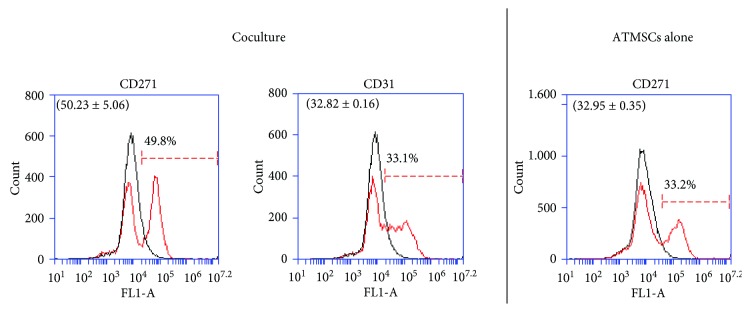
Flow cytometry histograms depicting cells from coculture of ATMSCs and HUVECs in ECM as spheroids and ATMSCs cultured alone in ECM as spheroids. After coculturing ATMSCs and HUVECs in ECM as spheroids, 49.8% of cells were found to express CD271 and 33.1% of cells expressed CD31. When cultured alone in ECM as spheroids, 33.2% of ATMSCs expressed CD271. Histograms drawn in black represent negative controls. Histograms drawn in red represent the expression of the abovementioned molecules by the cell populations. Overlapping curves represent populations of cells that do not express the tested markers. These data are representative of four independent experiments. Mean ± standard deviations of the percentage of cells expressing the surface markers are indicated in parenthesis.

ATMSCs were cocultured with HUVECs as spheroids produced by culturing cells in suspension on low attachment surfaces, which leads to the formation of cell aggregates [46]. Although other coculture systems that allow interaction between pericytes and ECs in extracellular matrices such as collagen are available [47], this spheroid coculture system was chosen because of its ease of implementation and low cost. The spheroids were formed by mixing a total of 500,000 cells at a ratio of 2 : 3 (ATMSCs : HUVECs) and culturing them in polypropylene tubes. ATMSCs were cultured in PCM for 7 days prior to coculture with HUVECs as spheroids in ECM for additional 7 days. Then, individualized cells from the spheroids were subjected to flow cytometry. We found that about 50% of cells expressed the nPC marker CD271 (Figure 4). The endothelial cell marker CD31 was detected in 33% of cells (Figure 4). We attribute the lower percentage of CD31-expressing cells observed after the flow cytometry analysis to the loss of HUVECs during the spheroid processing for flow cytometry, which is particularly harsh on HUVECs especially because, in spite of the action of collagenase, some mechanical disaggregation by pipetting is required for cell dissociation. As a control, we cultured ATMSCs for 7 days in PCM before culturing them alone as spheroids for additional 7 days. We found that about 30% of ATMSCs cultured alone in ECM as spheroids expressed the nPC marker CD271 (Figure 4). The preparation of HUVEC-alone spheroids was also attempted, but HUVECs failed to form spheroids alone—only clusters with a few cells developed under the conditions used in this study (not shown). Additionally, these small cell clusters were not dissociable by collagenase digestion even with some physical disaggregation by pipetting, which forced us to remove these controls from the analyses.

**Figure 5:**
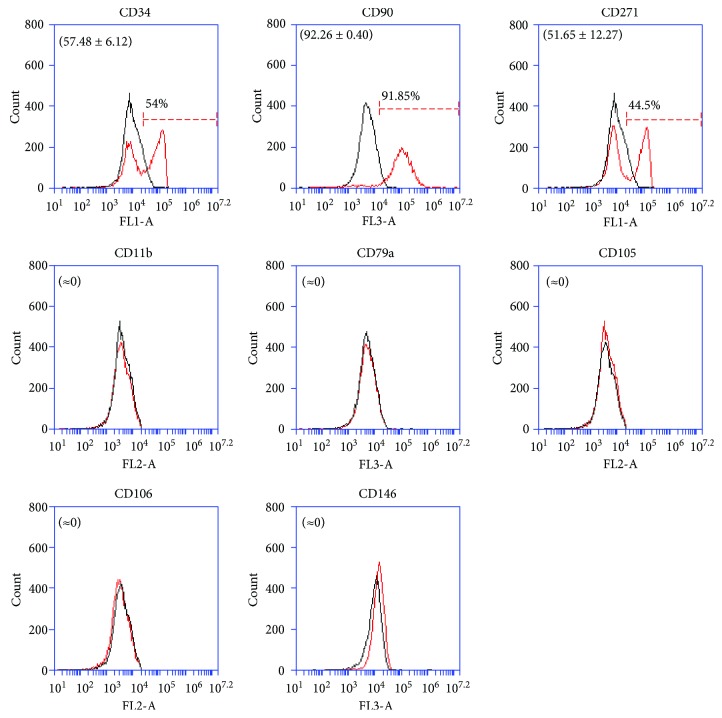
Flow cytometry histograms of ATMSCs cultured as spheroids in PCM for 7 days. Cells expressed CD34, CD90, and CD271 but did not express CD11b, CD79a, CD105, CD106, and CD146. Histograms drawn in black represent negative controls. Histograms drawn in red represent the expression of the abovementioned molecules by the cell populations. Overlapping curves represent populations of cells that do not express the tested markers. These data are representative of four independent experiments. Mean ± standard deviations of the percentage of cells expressing the surface markers are indicated in parenthesis.

When spheroids were formed in ECM, the objective of using this medium was to make sure HUVECs, which are more delicate than ATMSCs, would remain functional during the experiments. Spheroids formed by ATMSCs alone in ECM were meant to serve as negative controls. The realization that HUVECs were not the key factor for the expression of CD271 by ATMSCs led to the use of PCM for subsequent experiments as ATMSCs respond well to this medium, which is simpler to assemble than ECM. When flow cytometry analysis was performed on the individualized cells from the spheroids formed by ATMSCs alone in PCM, which were expected to work as negative controls, we found the expression of the nPC markers CD34 and CD271 in 54 and 44.5% of cells, respectively (Figure 5). Since no CD34^+^ or CD271^+^ cells were present in any of the ATMSC populations cultured in PCM in 2D conditions (Figure 2) and the time under spheroid culture conditions was short (one week), amplification of small populations of preexisting CD34^+^ or CD271^+^ cells is unlikely. CD146 was not detected on the surface of cells from the spheroids formed by ATMSCs alone (Figure 5). In a previous work by our group, freshly isolated pericytes did not express this molecule on their surface [17], a finding that was attributed to cleavage of the CD146 ectodomain by matrix metalloproteinase 3 (MMP3) [48]; recently, production of MMP3 by perivascular CD271^+^ cells in human synovial tissue was confirmed [49].

**Figure 6:**
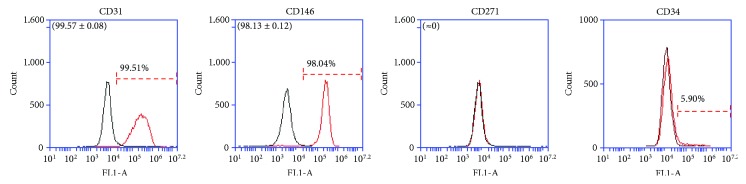
Immunophenotyping of HUVECs cultured in standard conditions (ECM, 2D). Virtually, all cells expressed CD31 and CD146, but none expressed of CD271 ((a); histograms representative of three independent experiments). When the expression of CD34 was assessed in a single HUVEC population (b), only 5.9% of the cells were found to be positive. Histograms drawn in black represent negative controls. Histograms drawn in red represent the expression of the abovementioned molecules by the cell populations. Overlapping curves represent populations of cells that do not express the tested markers. Mean ± standard deviations of the percentage of cells expressing the CD31, CD146, and CD271 are indicated in parenthesis.

The expression of other MSC markers was then assessed in ATMSCs derived from spheroids formed in PCM, and these cells were found to express CD90 but not CD11b, CD79a, CD105, or CD106 (Figure 5). HUVECs cultured in standard conditions were positive for CD31 and CD146 but not CD271 (*n* = 3); in one population analyzed, only about 6% of these cells expressed CD34 (Figure 6). Finally, in a single experiment performed to verify their differentiation ability in vitro, CD271^+^ cells isolated from spheroids containing only ATMSCs were culture expanded and could be induced to differentiate along osteogenic and adipogenic pathways (not shown). When cultured, these CD271^+^ cells lost CD271 expression but retained expression of the ATMSC markers CD90 and CD105 (not shown) and could be passaged at least three times. No further experiments using these cells were performed.

### 3.4. Influence of Culture Conditions on Expression of CD34 and CD271 by ATMSCs

**Figure 7:**
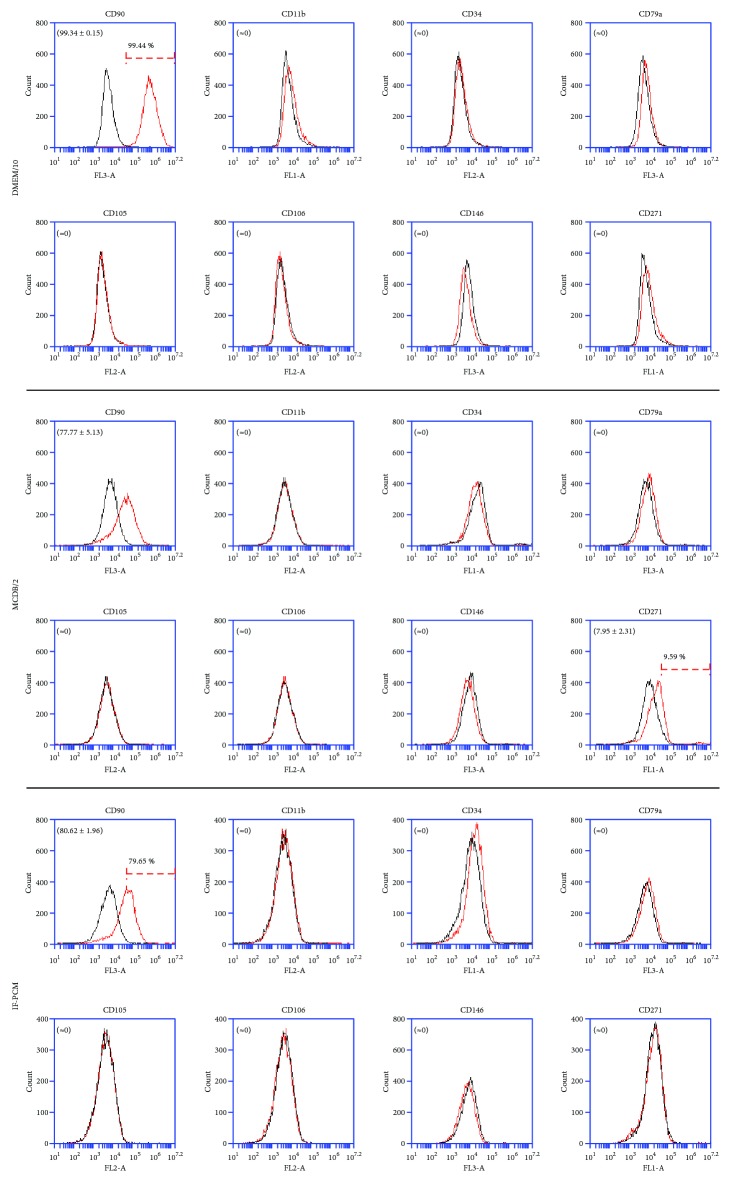
Immunophenotyping of ATMSCs from spheroids cultured in DMEM/10, MCDB/2, or IF-PCM for 7 days. Cells expressed CD90 but lacked expression of CD11b, CD34, CD79a, CD105, CD106, CD146, and CD271. A minor percentage of ATMSCs from spheroids cultured in MCDB/2 for 7 days also expressed CD271. Histograms drawn in black represent negative controls. Histograms drawn in red represent the expression of the abovementioned molecules by the cell populations. Overlapping curves represent populations of cells that do not express the tested markers. These data are representative of two independent experiments. Mean ± standard deviations of the percentage of cells expressing the surface markers are indicated in parenthesis.

To verify the influence of culture conditions on the expression of nPC and MSC markers in ATMSCs from spheroids, we performed flow cytometry of ATMSCs subjected to various culture conditions prior to culture as spheroids. Firstly, we cultured ATMSCs as spheroids for 7 days in DMEM/10. When these cells were analyzed by flow cytometry, they exhibited a classical MSC immunophenotype, except that CD105 expression was lacking (Figure 7). ATMSCs were also cultured with MCDB/2 (PCM without growth factors) for 7 days and then as spheroids for 7 more days in MCDB/2. Flow cytometric analysis of these cells showed that cells present a classical mesenchymal phenotype (Figure 7), with no expression of CD34 but with a slight expression of CD271. Finally, we precultured ATMSCs for 7 days in IF-PCM (PCM with no added IGF-1) and then cultured them as spheroids for 7 days. We chose to remove IGF-1 from the culture medium because of the well-known effects of this growth factor on proliferation and differentiation in a broad range of cell types [50]. After flow cytometric analysis of individualized cells from spheroids cultured in IF-PCM, we found the expression of the classical MSC marker CD90, and absence of CD11b, CD34, CD79a, CD105, CD106, CD146, and CD271 (Figure 7).

## 4. Discussion

Several studies have suggested MSCs can become pericytes when cocultured with vascular-like structures formed by ECs [20–25, 27–30], but pericyte markers used in those studies are already present on MSCs. CD271 and CD34 are expressed by noncultured human AT pericytes, and their expression is lost by these cells after expansion in culture, which indicates they are specific to mature pericytes [17]. Therefore, presence of these molecules on the surface of ATMSCs was used as an endpoint to assess the acquisition of a phenotype reminiscent of nPCs by MSCs cocultured with ECs. Pilot experiments demonstrated increased expression of *CSPG4*, *NGFR*, and *CD34* (genes that code for NG2, CD271, and CD34, resp.) in ATMSCs that were cocultured with murine ECs on Matrigel (Supplementary Table 3). In these pilot experiments, we also observed the colocalization of a fraction of ATMSCs with tubular structures formed by ECs on Matrigel, which is a feature of pericytes (Supplementary Figure 1).

Expression of CD34 by MSCs, pericytes, and other postnatal multipotent cells has been discussed through the past few years [51]. Another type of stromal cell, the telocyte, has been shown to be positive for CD34, and to acquire expression of *α*-SMA *in vivo* during tissue repair in the enteric wall, acting as a progenitor cell [52]. The vasa vasorum of the human saphenous vein [53] and the human neonatal heart [54] contain CD34^+^ pericytes. Additionally, several studies have reported that cells expressing CD34, but not CD31 and CD146, in the tunica adventitia of AT blood vessels are able to give rise to cells with MSC characteristics in vitro [55–59]. In these works, the authors considered expression of CD146 and lack of expression of CD31 and CD34 as characteristics of nPCs. However, as reviewed by Lin and Lue [60], in vivo identification and discrimination of pericytes and MSCs in the AT is not an easy task. Histological analysis of AT indicates that cells in the tunica adventitia and endothelium of arteries and arterioles express CD34; the layer between the adventitia and endothelium contains cells that simultaneously express *α*-SMA and CD140b [61] or *α*-SMA and CD146 [55, 57]. Traktuev et al. [35] showed that CD34^+^ cells obtained from AT also express the pericyte markers NG2, CD140a, and CD140b, are located in the perivascular region in vivo, and interact with ECs to form tubular structures in vitro. Furthermore, Braun et al. [62] identified a population of stromal cell-like cells that express CD34 and CD271, but not CD146, and give rise to cultured MSCs, unlike CD146-expressing perivascular cells, which do not express CD34. In a previous study published by our group, pericytes were isolated from human AT using an elaborate process; these noncultured pericytes were found to be positive for CD34 and CD271 while negative for CD146 and essentially become MSCs after expansion in culture [17].

Cattoretti et al. were the first to demonstrate expression of CD271 by human bone marrow pericytes (adventitial reticular cells) in the human bone marrow stroma [36]. More recently, Madelung et al. [63] and Flores-Figueroa et al. [37] also detected CD271 on human bone marrow adventitial reticular cells. Sowa et al. [64] identified CD271^+^ cells aligned with blood vessels in the subcutaneous AT of mice and named them neural crest-derived adipose MSCs. Yang et al. [65] found expression of CD271 on cells in the vasa vasorum of the venous adventitia and in a limited population of cells in the adventitia of small vessels in AT. In human fetal liver, CD271 is present on cells with characteristics of liver pericytes (hepatic stellate cells (HSCs)) identified as positive for CD34 but negative for CD45 [66]. Interestingly, murine hepatic HSCs express CD271, and addition of its ligand (nerve growth factor (NGF)) increases the frequency of apoptotic HSCs in serum-free culture [67]. Since no evidence of a significant amount of apoptosis in the CD271^+^ cells in the spheroids was present, we did not investigate apoptosis in these cells. CD271 expression has also been shown to be induced in ECs by exposure to high glucose, leading to release of microRNA-503, which negatively affects pericyte function [68]. Clearly, the HUVECs used in this study did not express CD271, nor did any of the media used by us contain a high glucose content—the amount of glucose in MCDB-131 or DMEM is 1.0 g/L, which represents a physiological glucose concentration.

MSC cultures established from CD271^+^ cells isolated from human bone marrow [69] or AT [70] are more proliferative, more clonogenic, and have a greater capacity to differentiate into mesodermal lineages than their CD271^−^ counterparts. MSC cultures established from CD271^+^ bone marrow cells also produce more cytokines, have greater immunosuppressive effects, and promote greater engraftment of hematopoietic cells in vivo than MSCs selected by adherence to plastic [71]. These findings indicate that MSCs obtained from CD271^+^ cells have greater potential for therapeutic applications. In this context, acquisition of CD271 expression by ATMSCs may not only suggest the acquisition of a pericytic phenotype by them, as it may also indicate occurrence of a transition to a more primitive, plastic state. Consequently, culture conditions that promote CD271 expression on MSCs could help counteract the long-known loss of multipotency in these cells after several passages in culture [72]. Comparing the clonogenicity and differentiation ability between CD271^+^ and CD34^+^ cells that developed in the spheroids and ATMSCs established using traditional methods was beyond the scope of this study; therefore, further studies are warranted to explore the full potential of these cells

Since no other studies have reported the acquisition of an nPC-like phenotype by MSCs without cell contact with ECs, one question arises. If it is not the interaction with ECs that makes MSCs acquire an nPC phenotype, what drives this conversion? Two hypotheses come up supported by our findings: interaction between cells in a 3D environment and presence of IGF-1 in the culture medium.

Culturing MSCs as spheroids has already been pointed as an efficient manner to improve their stemness, immunomodulatory capacity, and angiogenic effects [46, 73]. Gene expression is altered in MSCs cultured as spheroids, which includes upregulation of anti-inflammatory [74–76] and cell-cell/cell-extracellular matrix interaction genes [77, 78]. Therefore, the 3D microenvironment provided by the spheroid seems to improve cell communication and mimic characteristics of the in vivo pericyte niche, which may coax MSCs to take up an nPC-like phenotype.

IGF-1 exerts a broad range of physiological effects on the vasculature by means of both endocrine and autocrine/paracrine mechanisms [79]. IGF-1 stimulates the expression of VEGF [80, 81], which is a potent mitogen for ECs and plays important roles in angiogenesis [15]. Interestingly, IGF-1 has been shown to favor the development of CD31^−^CD34^+^CD146^−^ cells at the expense of development of CD31^−^CD34^+^CD146^+^ cells in AT [82]. In line with this finding, no CD146^+^ cells were detected by us in spheroids formed by MSCs alone in the presence of IGF-1, a condition that allowed expression of CD34 and CD271 by MSCs. Since expression of these markers was not appreciable in spheroids lacking added IGF-1, we hypothesize that this growth factor, along with the microenvironment provided by the spheroid, plays major roles in the conversion of MSCs into pericyte-like cells. Recently, *IGF-1* was found to be distinctively expressed by noncultured human AT pericytes but not expressed by culture-expanded pericytes [40]. Together, these data suggest that IGF-1 contributes to the acquisition and maintenance of a pericytic phenotype, whether in a paracrine or autocrine way. Since this study was not designed to examine the effects of IGF-1 on the acquisition of an nPC-like phenotype by ATMSCs, the question as to whether the effects of this growth factor on the expression of CD34 or CD271 by ATMSCs are dose-dependent remains unanswered.

## 5. Conclusions

Defining ways to improve stemness of MSCs is of special interest in the field of regenerative medicine, in which these cells are expected to be important tools. In addition to being a marker for a subset of pericytes in various human tissues, CD271 is a molecule associated with multipotency. Previously published reports indicate that MSCs derived from CD271^+^ cells exhibit superior proliferation, differentiation, and immunosuppressive/trophic abilities as compared to MSCs derived from CD271^−^ cells. Therefore, the culture conditions described herein may be used to provide MSCs with a greater ability to serve therapeutic purposes. Our results show, for the first time, that expression of CD271 and CD34 in ATMSCs can be effected in vitro using specific conditions in the absence of endothelial cells.

## Figures and Tables

**Table 1:** Description of the culture media used in the experiments.

Acronym	Culture medium description	Application
DMEM-LB/2	Low-glucose Dulbecco's Modified Eagle's Medium supplemented with 2.2 g/L of sodium bicarbonate (i.e., low bicarbonate), 10 mM HEPES, and 2% of FBS.	Collagenase dilution.
DMEM/10	Low-glucose Dulbecco's Modified Eagle's Medium supplemented with 3.7 g/L of sodium bicarbonate, 10 mM HEPES, 1% of penicillin/streptomycin solution, and 10% of FBS.	Culture of ATMSCs (2D and as spheroids). Basal medium for differentiation induction media.
MCDB/2	MCDB 131 supplemented with 2% FBS and 1% of penicillin/streptomycin solution.	Culture of ATMSCs (2D and as spheroids). PCM basal medium.
PCM	Pericyte medium—MCDB 131 supplemented with 2% FBS and 1% of penicillin/streptomycin solution, 2 ng/mL of EGF, 2 ng/mL of FGFb, 2 ng/mL of IGF-1 (LONG®R^3^ IGF-I), 5 *μ*g/mL of insulin from bovine pancreas and 1 *μ*g/mL of hydrocortisone.	Culture of ATMSCs (2D and as spheroids).
IF-PCM	IGF-1-free PCM.	Culture of ATMSCs as spheroids.
ECM	Endothelial cell medium—MCDB 131 supplemented with 2% FBS and 1% of penicillin/streptomycin solution, 10 ng/mL of EGF, 5 ng/mL of FGFb, 20 ng/mL of IGF-1, 1 *μ*g/mL ascorbic acid 2-phosphate, 10 IU/mL of heparin and 0.2 *μ*g/mL of hydrocortisone.	Culture of HUVECs (2D) and coculture of ATMSCs with HUVECs as spheroids.

## Data Availability

The data used to support the findings of this study are available from the corresponding author upon request.

## Supplementary Materials

Supplementary Materials The supplementary material file contains the following information: Supplementary methods for the culture of cells Matrigel; Supplementary Figure 1: ATMSCs cocultured with EOMA cells for 7 days on Matrigel. Supplementary Table 1: antibodies used to detect surface markers in this study. Supplementary Table 2: real-time PCR gene expression analysis of *NGFR*, *CSPG4*, and *CD34* in ATMSCs, EOMA cells, or ATMSCs cocultured with EOMA cells in 2D or on Matrigel. Supplementary Table 3: assessment of the expression of *NGFR*, *CSPG4*, and *CD34* by real-time PCR in ATMSCs after culture under various conditions.
