# Supplementary material for: Induction of Expression of CD271 and CD34 in Mesenchymal Stromal Cells Cultured as Spheroids
Source: Stem Cells Int. 2018 Jul 31;2018:7357213. doi: 10.1155/2018/7357213 (PMC6091361; doi:10.1155/2018/7357213)
Supplement: Supplementary Materials — The supplementary material file contains the following information: Supplementary methods for the culture of cells Matrigel; Supplementary Figure 1: ATMSCs cocultured with EOMA cells for 7 days on Matrigel. Supplementary Table 1: antibodies used to detect surface markers in this study. Supplementary Table 2: real-time PCR gene expression analysis of NGFR, CSPG4, and CD34 in ATMSCs, EOMA cells, or ATMSCs cocultured with EOMA cells in 2D or on Matrigel. Supplementary Table 3: assessment of the expression of NGFR, CSPG4, and CD34 by real-time PCR in ATMSCs after culture under various conditions. [file 7357213.f1.pdf]

Supplementary Material

Supplementary Methods

Culture of cells on Matrigel®

ATMSCs were cultured alone or in a coculture system with the murine EC lineage EOMA (Rio de Janeiro Cell Bank, Rio de Janeiro, RJ, Brazil) on Matrigel® (Becton, Dickinson and Company). After coating 12-well culture plates with Matrigel®, cells were seeded at a density of 1.5×10^5 cells per well when cultured alone, or 1.5×10^5 EOMA cells plus 4×10^4 ATMSCs when cocultured. EOMA cells and ATMSCs were stained intracellularly with CM-DiI or DiO (Molecular Probes, Invitrogen™, Eugene, OR, USA), respectively, following the manufacturer's instructions. Vital staining was chosen to observe the behavior of cells during the cultivation. Cocultured cells were analyzed on an Axio Observer Z1 inverted microscope using filter sets 43HE and 38HE (Carl Zeiss, Jena, Germany), respectively. Images were acquired using a digital camera coupled to the microscope (AxioCam MRm Rev.3; Zeiss).

RNA extraction and cDNA synthesis

After culturing cells on Matrigel®, total RNA was extracted using TRIzol® (Life Technologies Corporation, Carlsbad, CA, USA), following the manufacturer's instructions. Synthesis of cDNA was carried out using M-MLV reverse transcriptase enzyme and hexameric random primers from Applied Biosystems (Foster City, CA, USA) following the manufacturer's instructions.

Real-time PCR

Real-time PCR reactions were performed in a Step One Plus termocycler (Applied Biosystems). We used inventoried TaqMan® (Applied Biosystems) gene expression assays to assess the expression of the following genes: *GAPDH* (Assay ID: Hs02786624_g1), *NGFR* (Assay ID: Hs00609977_m1), *CSPG4* (Assay ID: Hs00361541_g1), and *CD34* (Assay ID: Hs00990732_m1). Results are presented as ΔCT values for each gene in relation to the endogenous control, *GAPDH*. Comparisons were performed using the $2^{-\Delta\Delta CT}$ formula, where ΔCT values from different culture conditions were subtracted in the comparisons.

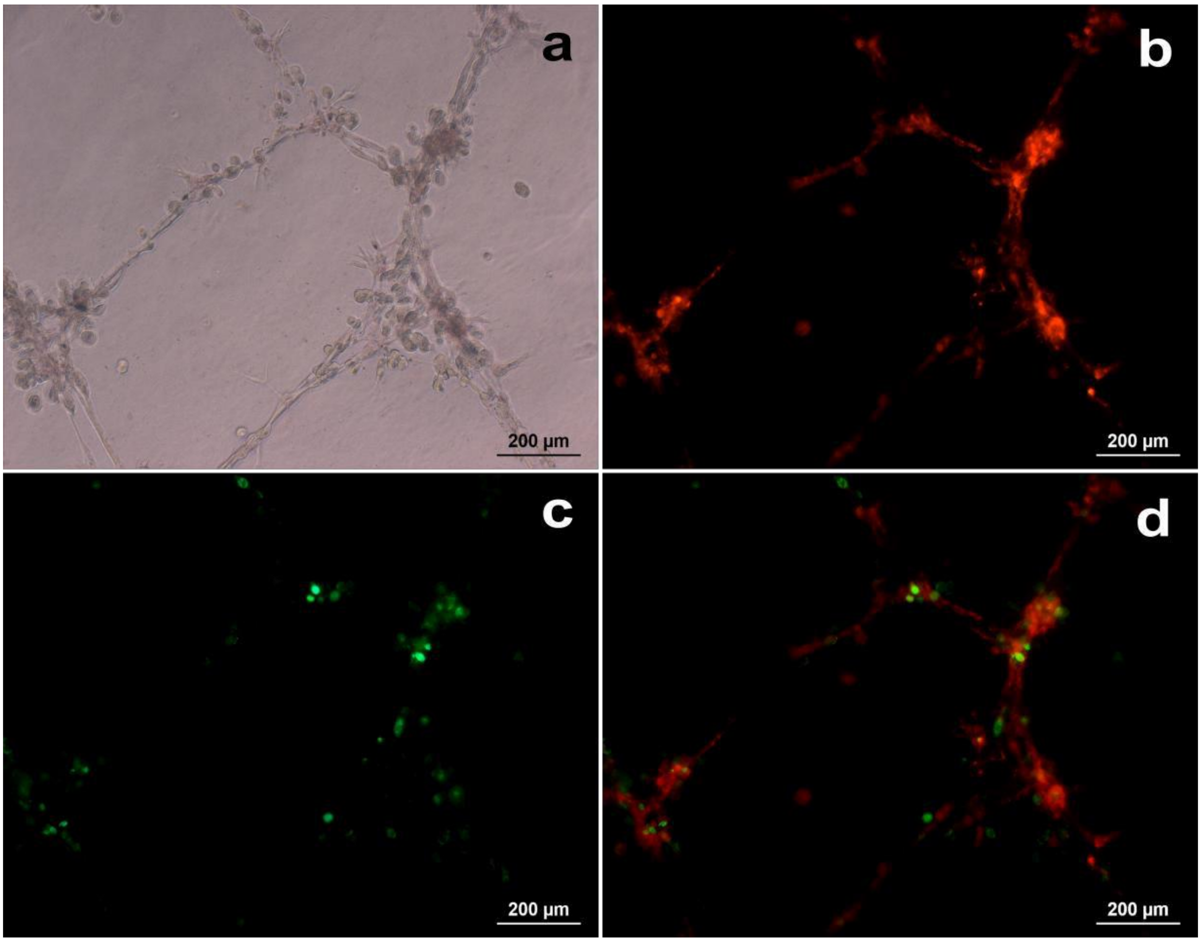

Supplementary Figure 1: ATMSCs cocultured with EOMA cells for 7 days on Matrigel®. (a) Phase-contrast image of the coculture on Matrigel®. (b) Epifluorescence image of the same field depicted in (a); EOMA cells are shown in red owing to previous intracellular staining with CM-DiI. (c) Epifluorescence image of the same field depicted in (a) and (b); ATMSCs are shown in green owing to previous intracellular staining with DiO. (d) Composite image made by merging the images shown in (b) and (c).

Supplementary Table 1: Real-time PCR gene expression analysis of *NGFR*, *CSPG4,* and *CD34* (which encode CD271, NG2, and CD34, respectively) in ATMSCs, EOMA cells, or ATMSCs cocultured with EOMA cells in 2D or on Matrigel®. Values are shown as ΔCT for each gene.

	**ΔCT**		
**Samples**	***NGFR***	***CSPG4***	***CD34***
ATMSCs cultured for 3 days (2D)	12.53	6.58	10.33
EOMA cultured for 3 days (2D)	nd	nd	nd
Coculture for 3 days (2D)	16.37	11.44	nd
ATMSCs cultured on Matrigel® for 3 days	11.39	6.48	nd
EOMA cultured on Matrigel® for 3 days	nd	nd	nd
Coculture on Matrigel® for 3 days	15.83	10.24	nd
ATMSCs cultured on Matrigel® for 7 days	9.46	6.58	10
EOMA cultured on Matrigel® for 7 days	nd	nd	10.88
Coculture on Matrigel® for 7 days	6.29	3.58	7.4

nd: not detected

Supplementary Table 2: Assessment of the expression of *NGFR*, *CSPG4*, and *CD34* in ATMSCs after culture under various conditions using the $2^{-\Delta\Delta CT}$ formula. Each value represents fold-change increase in gene expression from the first condition relative to the second condition in the comparison.

	**$2^{-\Delta\Delta CT}$**		
**Conditions compared**	**NGFR**	**CSPG4**	**CD34**
1 - ATMSCs cultured for 3 days (2D)<br>2 - Coculture for 3 days (2D)	14.32	29.04	a
1 - ATMSCs cultured on Matrigel® for 3 days<br>2 - ATMSCs cultured for 3 days (2D)	2.20	1.07	b
1 - ATMSCs cultured on Matrigel® for 7 days<br>2 - ATMSCs cultured on Matrigel® for 3 days	3.81	0.93	a
1 - Coculture on Matrigel® for 3 days<br>2 - Coculture for 3 days (2D)	1.45	2.30	nd
1 - Coculture on Matrigel® for 7 days<br>2 - ATMSCs cultured on Matrigel® for 7 days	9	8	6.06
1 - Coculture on Matrigel® for 7 days<br>2 - Coculture on Matrigel for 3 days	744.43	101.12	a

nd: fluorescence not detected in any of the conditions
a: gene expression detected in the first condition but not in the second condition
b: gene expression detected in the second condition but not in the first condition
